# The Role of Cellular Senescence in Oral Health and Disease

**DOI:** 10.3390/ijms27052269

**Published:** 2026-02-28

**Authors:** Mahmoud Elashiry, Celine Joyce Cornelius Timothius, Rizwana Zaman, Marisa Elliott, Bryce Crosby, Keshav Bhat, Karim M. Saad, Ranya Elsayed

**Affiliations:** Periodontics Department, Dental College of Georgia, Augusta University, Augusta, GA 30912, USA; melashiry@augusta.edu (M.E.); rzaman@augusta.edu (R.Z.); ksaad@augusta.edu (K.M.S.)

**Keywords:** cellular senescence, senescence-associated secretory phenotype (SASP), oral senescence, periodontal disease, alveolar bone loss, oral mucosal aging, inflammaging, chronic inflammation

## Abstract

Cellular senescence is a fundamental biological process characterized by stable cell cycle arrest, resistance to apoptosis, and the acquisition of a pro-inflammatory senescence-associated secretory phenotype (SASP). While senescence plays essential roles in development, tissue homeostasis, and tumor suppression, its accumulation with age and chronic stress contributes to tissue dysfunction and disease. In the oral cavity, where tissues are continuously exposed to mechanical stress, microbial challenge, and environmental insults, senescence has emerged as a critical regulator of both health and pathology. This review provides an overview of the defining hallmarks of cellular senescence, the molecular mechanisms driving its onset, and its physiological and pathological consequences, with a particular focus on oral tissues. We highlight the beneficial roles of senescence in maintaining oral tissue integrity, facilitating wound repair, suppressing malignant transformation, and promoting immune-mediated clearance of damaged cells. Conversely, we discuss the detrimental effects of persistent senescent cell accumulation, including oral aging phenotypes, chronic inflammation, alveolar bone loss, periodontal breakdown, salivary gland dysfunction, and contributions to oral carcinogenesis. Finally, we examine emerging therapeutic strategies targeting senescence in oral disease management, including senolytic and senomorphic approaches, immune-based clearance mechanisms, and gene- and cell-based interventions aimed at delaying or modulating senescence. Understanding the dualistic nature of cellular senescence in the oral environment may inform novel preventive and therapeutic strategies to promote oral health and mitigate age- and disease-associated oral pathologies.

## 1. Introduction

### 1.1. Defining Cellular Senescence and Its Biological Hallmarks

As advances in biomedical science and healthcare continue to extend human lifespan, survival rates in developed countries have risen steadily. By 2060, the U.S. population aged 65 and older is projected to reach nearly 95 million. This demographic shift is accompanied by a substantial increase in age-related conditions, including Alzheimer’s disease, cardiovascular disease, diabetes, and stroke [1]. The growing prevalence of these disorders carries profound economic consequences; for instance, in 2020, approximately 5% of all medical expenditures in the United States were attributed to Alzheimer’s disease and related dementias [2]. As aging populations expand, understanding the biological mechanisms that drive age-associated pathology and identifying effective therapeutic strategies has become increasingly critical. Among these mechanisms, cellular senescence has emerged as a key contributor not only to systemic aging and chronic disease but also to oral health and disease. The clinical significance of cellular senescence in oral health is becoming increasingly evident, underscoring the need for a deeper understanding of its causes, mechanisms, and therapeutic implications.

As the body ages, cells are increasingly exposed to a range of environmental and physiological stressors that threaten their integrity. In response to such damage, cells employ several protective mechanisms to prevent tumorigenesis and preserve tissue function. One such pathway is cellular senescence (from the Latin *senex*, meaning “old”), a state defined by a permanent arrest of the cell cycle in which cell division ceases [3]. The first evidence of this phenomenon was reported by Hayflick and Moorhead in 1961, when they observed that human fibroblasts undergo only a finite number of divisions, a finding that later became known as the “Hayflick limit” [4].

Senescent cells exhibit a range of biological hallmarks that distinguish them from proliferating cells, including characteristic morphological alterations. Traditionally, senescent cells have been described as enlarged and flattened in appearance [5,6]. However, recent work has expanded this view by identifying a broader spectrum of morphological changes across multiple cellular compartments, collectively termed senescence-associated morphological phenotypes (SAMPs) [7]. In their work, Wallis and colleagues advanced this concept by developing a high-content, image-based profiling method that quantifies 62 morphological features, including cell size, nuclear intensity, shape, and texture, to characterize senescence across diverse models induced by oncogenic stress, paracrine signaling, replicative exhaustion, and DNA damage. This approach effectively discriminated senescent from proliferating cells, revealing both inter-model heterogeneity (differences between senescence-inducing stimuli) and intra-model heterogeneity (variability among individual cells within the same model). Through exploratory factor analysis, the authors distilled these features into a smaller set of biologically meaningful latent factors. Notably, SAMPs were also applicable in vivo, as demonstrated in palbociclib-treated human tumor xenografts, where senescent (p21-positive) and non-senescent cells exhibited distinct morphological signatures. Collectively, these findings position SAMPs as a valuable first-pass or complementary tool for the identification and characterization of senescence, broadening the phenotypic framework beyond traditional markers.

In addition to their characteristic morphological changes, senescent cells display numerous molecular hallmarks related to genomic integrity and gene expression. Activation of the DNA damage response (DDR) is a critical decision point that determines whether a cell will repair the damage and resume normal function, undergo apoptosis, or enter senescence [8,9,10]. However, recent evidence from *Drosophila* suggests that apoptosis resistance is a prerequisite for senescence induction, rather than a downstream consequence, positioning it as a defining feature of senescent cells [11]. Once the DDR is activated, it initiates signaling cascades that increase the expression of the cyclin-dependent kinase (CDK) inhibitors p21 (via p53 activation) and p16, leading to retinoblastoma (Rb) pathway activation—key regulators of senescence induction. While both pathways contribute to enforcing cell-cycle arrest, p16–Rb is increasingly recognized as a key regulator of late-stage senescence, particularly in contexts where p53 expression is impaired [12,13,14]. These CDK inhibitors cooperate to enforce the permanent cell-cycle arrest that defines senescence. While DNA damage initiates senescence, it also accumulates during the senescent state, primarily due to increased oxidative stress and reduced expression of DNA repair genes.

Mitochondrial dysfunction is a hallmark of senescence, generating elevated reactive oxygen species (ROS) that promote DNA damage and sustain the DDR [15]. Senescent cells also exhibit downregulation of DNA repair genes, largely via p16–Rb-mediated inhibition of E2F transcription factors, leading to persistent DDR activation, a hallmark of senescence [8,9]. γ-H2AX, the phosphorylated form of histone H2AX at serine-139, accumulates at sites of DNA double-strand breaks. In normal proliferating cells, γ-H2AX recruits DNA repair machinery; however, in senescent cells with impaired repair capacity, these foci accumulate, serving as robust indicators of DNA damage and senescence [16,17,18]. In addition to γ-H2AX, senescence is characterized by extensive chromatin remodeling. Senescence-associated heterochromatic foci (SAHF) are dense, transcriptionally repressive regions of chromatin that accumulate within the nuclei of senescent cells. SAHFs play a key role in silencing genes that drive proliferation, including E2F target genes, thereby reinforcing stable cell-cycle arrest [19].

The most prominent hallmark of senescence—and a major contributor to many age-related diseases—is the Senescence-Associated Secretory Phenotype (SASP). Upon entering senescence, cells adopt a hypersecretory state, releasing a complex mixture of pro-inflammatory cytokines (e.g., IL-1α, IL-6, TNF-α), chemokines (CXCLs and CCLs), matrix-modifying enzymes (e.g., MMPs), ROS, and extracellular vesicles, including exosomes [20,21,22]. Notably, senescent cells show a marked increase in exosome production, which serves as an important mechanism for intercellular communication. These exosomes carry proteins, nucleic acids, and microRNAs that can propagate senescence signals, modulate the tissue microenvironment, and influence immune responses, amplifying the effects of the SASP [23,24,25]. The composition of the SASP is highly heterogeneous, depending on cell type and the specific trigger of senescence [20,22]. Under physiological conditions, SASP factors aid in the clearance of aged or potentially malignant cells by the immune system [26,27,28]. However, persistent accumulation of senescent cells leads to chronic SASP exposure, and the elevated release of exosomes can exacerbate inflammation and tissue dysfunction, making the SASP a central driver of many age-related diseases (discussed further in Section 2.1).

Senescent cells also display heightened lysosomal activity, with constitutive activation of both macro-autophagy and chaperone-mediated autophagy [29]. Elevated lysosomal β-galactosidase activity at pH 6, detected by the widely used SA-β-Gal assay, serves as a reliable marker of senescence [30,31]. Beyond serving as a biomarker, autophagy functionally supports senescence: the amino acids generated by autophagic degradation activate mTOR signaling, a central regulator of metabolism that fuels SASP production and secretion [32,33]. This mechanistic link between lysosomal function, autophagy, and SASP underscores how metabolic remodeling sustains the pro-inflammatory and tissue-modifying features of senescent cells.

Collectively, the hallmarks of senescence, including persistent DDR, chromatin remodeling (e.g., SAHF, γ-H2AX), SASP, mitochondrial dysfunction, and lysosomal activation, highlight its complexity and central role in tissue aging and disease [34]. Each of these features contributes to the stable cell-cycle arrest, pro-inflammatory secretome, and metabolic remodeling that define senescent cells, yet no single marker is sufficient for definitive identification. Reliance on individual features, such as SA-β-Gal activity or γ-H2AX accumulation, can lead to misinterpretation of data and over- or underestimation of senescent cell burden. This limitation underscores the need for a comprehensive atlas of tissue-specific senescence markers in both mice and humans, enabling more precise characterization of senescent cells across tissues, contexts, and disease states. Establishing such a framework is particularly relevant for oral health, where accurate identification of senescent cells will be critical to understanding their contributions to chronic inflammation, tissue degeneration, and age-associated oral pathologies, and to developing targeted therapeutic strategies.

It is important to acknowledge that documenting cellular senescence in vivo remains technically challenging. Senescence is a heterogeneous and dynamic cellular state, and no single biomarker definitively identifies senescent cells across tissues or disease contexts [34,35]. Commonly used indicators such as p16^INK4a^, p21^CIP1^, SA-β-gal activity, and selected SASP components may also be expressed during transient stress responses, inflammation (e.g., in macrophages), or differentiation, complicating interpretation in complex tissues [36]. As recently emphasized, robust identification of senescence in vivo requires combinatorial, spatial, and functional approaches rather than reliance on individual markers alone [34,37]. This need for rigor has culminated the development of MICSE [Minimal Information on Cellular Senescence Experimentation in vivo] guidelines, which provide an international standardized framework for the multiparametric validation of senescent cells in living organisms [38].

### 1.2. Mechanisms Leading to Senescence Onset

A wide range of senescence inducers has been identified and well documented in the literature. Among these, the most notable and well-characterized triggers include replicative stress, oncogene activation, oxidative stress, infection, irradiation, and chemotherapeutic agents. Less commonly highlighted, but highly relevant to oral health, are hyperglycemia-induced and mechanically induced senescence, both of which play essential roles in tissue aging and disease. The major inducers of cellular senescence in oral tissues and their dominant molecular pathways are summarized in Table 1.

#### 1.2.1. Replicative Stress

Replicative stress is a fundamental trigger of cellular senescence and is particularly relevant to tissues with high turnover, such as those in the oral cavity. It occurs when DNA damage interferes with replication fork progression, leading to genomic instability. A major contributor to replicative stress is telomere attrition, an inevitable consequence of repeated cell divisions. With each replication cycle, telomeric DNA shortens until a critical threshold is reached, activating the DDR pathway and driving cells into a senescent state [39]. Notably, replicative stress-induced senescence was the first form of senescence described in the literature, laying the foundation for our understanding of this process [4]. In oral tissues, where epithelial and immune cells undergo continuous renewal, replicative stress may play a key role in age-associated dysfunction and disease.

#### 1.2.2. Oncogene Activation

Oncogene-induced senescence (OIS) serves as a vital safeguard against malignant transformation. When oncogenes such as RAS or components of the MAPK pathway become activated, mitogenic signaling increases dramatically, driving uncontrolled cellular division that leads to replication stress, DNA damage, and telomere fragility. As this damage accumulates, the DDR pathway is activated, inducing stable cell-cycle arrest [40,41,42,43]. In the oral cavity, OIS helps prevent the progression of precancerous lesions to oral cancers [44]. In this context, senescent cells exhibit characteristic morphological changes and a SASP that can influence surrounding epithelial and stromal cells [45]. While OIS provides critical tumor-suppressive effects, persistent senescent cells and chronic SASP in oral tissues may contribute to local inflammation, tissue remodeling, and age-related oral pathologies, including periodontal disease, impaired wound healing, and increased susceptibility to neoplastic transformation [46].

#### 1.2.3. Oxidative Stress

Oxidative stress is both a trigger and a reinforcing factor for cellular senescence. It arises from the accumulation of ROS, which can cause significant DNA damage. As this damage accumulates, the DDR pathway is activated, leading to stable cell cycle arrest and senescence. Mitochondrial dysfunction, a hallmark of senescent cells, is a major source of intracellular ROS, further amplifying oxidative stress [15]. Thus, oxidative stress not only contributes to the onset of senescence but also helps maintain and reinforce the senescent phenotype, with important implications for oral tissues, including the aging oral epithelium, impaired wound healing, and increased susceptibility to periodontal disease.

#### 1.2.4. Chemotherapy-Induced Senescence

Chemotherapeutic agents activate the DDR pathway through drug-induced DNA lesions, triggering cellular senescence. While senescence effectively halts proliferation, the accumulation of senescent cells with a SASP can paradoxically promote malignant transformation in neighboring cells [47,48,49,50]. Indeed, chemotherapy-induced senescence (CIS) is recognized as a major contributor to chemotherapy resistance [51]. Notably, CIS has been observed in head and neck squamous cell carcinoma, highlighting its relevance as a senescence trigger in oral pathologies [52]. These findings underscore the dual role of senescence in oral health, functioning both as a tumor-suppressive mechanism and as a contributor to therapy resistance and disease progression.

#### 1.2.5. Irradiation

Radiation exposure, from ultraviolet (UV) light, environmental sources, or radiation therapy, has been shown to activate the DDR pathway, leading to cellular senescence [53,54]. Similar to the mechanisms described for replicative stress and oncogene activation, radiation compromises DNA integrity, resulting in the accumulation of senescent cells. In the context of oral tissues, this accumulation has been linked to adverse outcomes such as hyposalivation following head and neck radiation therapy, highlighting the impact of radiation-induced senescence on oral health and function [55].

#### 1.2.6. Infection

Infection-induced senescence is a less well understood but increasingly recognized trigger of cellular senescence. Certain viruses and bacteria can induce senescence through virulence factors that disrupt host cell processes and promote DNA damage or membrane fusion events [56]. For example, measles virus has been shown to induce senescence in human placental syncytiotrophoblasts through cell–cell fusion [57], while SARS-CoV-2 has been reported to drive senescence in human lung epithelial cells [58], endothelial cells [58], and fibroblasts [59]. These findings highlight the potential contribution of infection-induced senescence to age-dependent disease severity. In the context of oral health, infection-induced senescence plays a significant role in the pathogenesis of periodontal disease. Key periodontal pathogens, including *Porphyromonas gingivalis* and *Fusobacterium nucleatum*, can induce senescence in oral epithelial and gingival cells, thereby promoting chronic inflammation and contributing to both local periodontal breakdown and systemic inflammatory complications [60,61,62] (discussed in Section 3.2). This underscores the importance of infection-induced senescence as a mechanistic link between microbial challenge, oral tissue aging, and disease progression [60].

#### 1.2.7. Hyperglycemia

Although the mechanisms underlying hyperglycemia-induced senescence are not yet fully understood, increasing evidence indicates that elevated glucose levels can trigger premature senescence in multiple cell types. Hyperglycemia has been shown to induce senescence in human kidney tubular epithelial cells [63] and human endothelial cells [64,65,66], highlighting its impact on tissues vulnerable to metabolic stress. Importantly, hyperglycemia-induced senescence has also been observed in murine gingival macrophages [67], providing mechanistic insight into the well-recognized but poorly understood connection between diabetes and periodontal disease. Given the global prevalence of diabetes, further research is needed to clarify how chronic hyperglycemia drives senescence in oral tissues and how this contributes to periodontal inflammation, impaired healing, and long-term oral health complications.

#### 1.2.8. Mechanical Force

Mechanical forces exert a bidirectional influence on cellular senescence, with evidence supporting both pro-senescent and anti-senescent effects depending on the context. In murine models, mechanical unloading has been shown to promote the accumulation of senescent cells [68], suggesting a connection between reduced mechanical stimulation, immobilization, and the development of age-associated conditions. Conversely, studies using human tissue cultures demonstrate that increased mechanical stress, as found in fibrotic or “stiffened” tissues, can trigger senescence [69]. This dual nature of mechanical force has important implications for oral biology, particularly in orthodontic therapy (discussed in Section 3.2), where controlled mechanical loading can influence cellular aging, tissue remodeling, and the inflammatory response.

Within the oral cavity, where cells are continuously exposed to microbial challenges, metabolic fluctuations, mechanical loading, and environmental stressors, senescence acts as both a protective and pathogenic process. Early in life, senescence serves essential roles in tumor suppression, wound repair, and tissue homeostasis. However, with age and chronic exposure to stressors, senescent cells accumulate, and their persistent SASP contributes to a pro-inflammatory microenvironment, impaired regenerative capacity, and heightened susceptibility to disorders such as periodontitis, hyposalivation, and oral carcinogenesis. This duality mirrors the concept of evolutionary antagonistic pleiotropy, in which mechanisms that are beneficial for early-life fitness become detrimental in later life.

Understanding how these mechanisms operate and interact provides a critical foundation for examining the broader physiological and pathological implications of senescence in oral tissues, which will be the focus of the next section.

**Table 1:** Major Inducers of Cellular Senescence in Oral Tissues: Core Molecular Triggers and Dominant Pathways.

Senescence Inducer	Molecular Trigger	Senescence Pathway	Oral Impact	Reference
Replication stress/Telomere shortening	Progressive telomere shortening leading to uncapped chromosome ends	Telomere-driven DDR → p53/p21 signaling	Repeated turnover contributes to replicative senescence; contributes to epithelial thinning, reduced healing capacity and mucosal aging	[4,39]
Oncogene activation	Aberrant RAS/MAPK signaling induces replication stress	OIS-associated DDR → p16/Rb-dominant arrest	Premalignant epithelial stress	[40,41,42,43]
Oxidative stress	Excess ROS causes DNA strand breaks and mitochondrial damage	ROS-mediated DDR → p53/p21 ± p16/Rb	Redox-rich oral inflammatory environment	[15]
Chemotherapeutic agents	Drug-induced genotoxic stress and replication fork collapse	Therapy-induced senescence via p53/p21 and p16/Rb	Cancer treatment–associated tissue stress	[47,48,49,50,52]
Ionizing radiation (UV or therapeutic radiation)	Direct DNA double-strand breaks and ROS generation	Strong DDR → p53-dependent senescence	Head and neck radiotherapy exposure.	[53,55]
Periodontal bacterial infection (*P. gingivalis*, *F. nucleatum*)	Bacterial virulence factors, LPS, and OMVs induce DNA damage, mitochondrial dysfunction, and TLR signaling	Persistent DDR → p53/p21 and p16/Rb activation	Chronic microbial burden in periodontal tissues	[60,61,62]
Viral infection	Viral entry and replication induce DNA damage, cell–cell fusion, ER stress, and mitochondrial dysfunction	Virus-induced DDR with p53/p21 activation and NF-κB–driven senescence reinforcement	Persistent viral exposure in oral mucosa and salivary tissues	[57]
Hyperglycemia	Mitochondrial ROS overproduction and inflammasome priming	ROS-DDR coupling with inflammatory reinforcement	Diabetic oral microenvironment	[66,67]
Mechanical forces (loading/unloading)	Cytoskeletal disruption and altered mechano-transduction	Stress-activated p16/Rb signaling	Altered biomechanical forces in oral tissues	[68,70]

Major inducers of cellular senescence in oral tissues, their core molecular triggers, dominant senescence pathways, and associated oral impacts. Abbreviations: DDR, DNA damage response; OIS, oncogene-induced senescence; ROS, reactive oxygen species; LPS, lipopolysaccharide; OMVs, outer membrane vesicles; NF-κB, nuclear factor kappa B; p53/p21 and p16/Rb, canonical senescence regulatory pathways. References correspond to studies documenting each senescence inducer and its oral tissue effects.

## 2. Beneficial Aspects of Senescence in Oral Tissues

### 2.1. Role in Tissue Homeostasis and Repair

In oral tissues, transient, tightly regulated senescence supports homeostasis, wound healing, and regeneration by serving as a protective barrier against uncontrolled cell proliferation and genomic instability [71].

During development, senescence orchestrates proper morphogenesis [72,73]. In adult tissues, the temporary release of SASP factors recruits immune and progenitor cells to facilitate tissue repair and regeneration [74,75]. In oral tissues, controlled senescence maintains epithelial integrity, preserves cellular homeostasis, and prevents the proliferation of damaged or pre-malignant cells [26,27,28].

Following acute injury or microbial insult, oral epithelial cells, fibroblasts, endothelial cells, and immune cells can enter acute senescence, a temporary state that contributes to efficient tissue repair. During this phase, senescent cells release a regulated, pro-resolution form of the SASP, consisting of cytokines, chemokines, growth factors, and matrix-remodeling enzymes [76]. These factors orchestrate multiple repair processes: they promote fibroblast activation, enhance re-epithelialization, and recruit innate and adaptive immune cells, including neutrophils, monocytes, NK cells, and lymphocytes, to clear debris and eventually eliminate the senescent cells themselves. Evidence from acute cutaneous wound models demonstrates that the early accumulation of senescent fibroblasts and endothelial cells contributes to tissue repair processes. Mechanistically, these cells secrete specific SASP components, notably Platelet-Derived Growth Factor AA (PDGF-AA), which drive the differentiation of local mesenchymal cells into contractile myofibroblasts to facilitate wound closure. This beneficial, self-limiting secretory profile stands in sharp contrast to the persistent, unregulated pro-inflammatory SASP that drives chronic pathology and tissue dysfunction [77,78].

Senescent myofibroblasts also play an anti-fibrotic role by limiting excessive extracellular matrix deposition and promoting matrix turnover, thereby reducing scarring [79]. Additionally, senescent macrophages secrete IL-6, which enhances cellular plasticity and facilitates the transition of pro-inflammatory M1 macrophages toward the M2 pro-repair phenotype, supporting debris clearance and tissue remodeling [76,80,81]. Although direct evidence in oral wound healing is still emerging, these well-characterized mechanisms in cutaneous tissue strongly suggest that transient senescence also contributes to improved repair outcomes in oral mucosa and periodontal tissues. Understanding these beneficial roles may help guide the development of targeted regenerative strategies that harness the protective and pro-healing aspects of senescence while avoiding its chronic, pathological consequences.

### 2.2. Tumor Suppressive Function in the Oral Environment

While the molecular pathways underpinning senescence have been extensively studied in epithelial systems, comparatively fewer investigations have focused on senescence within the oral cavity. Bascones-Martínez et al. [42] evaluated markers associated with the ARF/p53/p21 pathway (p53, MDM2, Ki-67) and the p16/Rb/E2F pathway (Cyclin D1, Maspin, Rb) in normal, precancerous, and malignant oral tissues. Their findings demonstrated minimal senescence-associated expression in normal oral mucosa, increased expression in OSCC, and the highest levels in premalignant lesions. Similarly, a separate study of 50 oral leukoplakia cases, 32% of which exhibited dysplasia, reported universal p16^INK4a^ expression, with variable low (*n* = 30) and high (*n* = 20) expression levels, independent of HPV status [43]. Another investigation examining 86 oral epithelial dysplasia samples found progressive increases in Ki-67, γ-H2AX, and p53 expression with advancing dysplasia severity, followed by plateauing or reduction of these markers in OSCC [82].

Collectively, these studies suggest that senescence-associated pathways are more prominently activated in premalignant oral lesions than in either normal mucosa or overt carcinoma. This pattern supports the concept of senescence serving as an early tumor-suppressive barrier within the oral environment, potentially limiting malignant transformation during the initial stages of disease progression.

### 2.3. Immune Surveillance and Senescent Cell Clearance

SASP factors facilitate the recruitment of immune cells to support senescent cell clearance. Senescent cells secrete a range of cytokines and chemokines that attract macrophages, NK cells, and cytotoxic T cells, with the specific ligands produced varying across tissue microenvironments [83]. For example, SASP-mediated activation of CD4^+^ T cells has been shown to eliminate senescent premalignant hepatocytes [26], while senescent hepatic stellate cells in fibrotic liver upregulate NKG2D ligands that stimulate NK-cell-mediated clearance and contribute to fibrosis resolution [84].

Age-related declines in immune competency reduce the ability of CD8^+^ T cells and NK cells to eliminate abnormal or senescent cells, permitting their buildup. Immune evasion by senescent cells also becomes more prominent with age. Senescent cells can upregulate inhibitory immune-checkpoint ligands such as Programmed Death Ligand (PD-L1), which bind PD-1 receptors on CD8^+^ T cells and NK cells to dampen their cytotoxic activity. This evasion contributes to the persistence of senescent cells in aging tissues, including the oral mucosa, and may reinforce chronic inflammation and carcinogenic risk [85].

Together, these studies underscore that effective senescent cell clearance is not solely dictated by SASP composition but is critically constrained by immune competence and immune checkpoint signaling. Defining how these mechanisms operate in oral tissues remains an important gap in the field.

## 3. Detrimental Roles of Cellular Senescence in Oral Diseases

Increasing evidence indicates that senescent cells accumulate across a wide spectrum of oral disease, where they contribute to chronic inflammation, tissue degeneration, impaired regeneration, and malignant transformation in a disease- and tissue specific manner. An overview of senescent cell types, markers, SASP profiles and functional consequences in major oral diseases is shown in Table 2.

### 3.1. Senescence-Driven Oral Aging Phenotypes

Aging profoundly affects the oral mucosa through cumulative cellular alterations as well as environmental and systemic factors. Contributing influences include reduced salivary flow, chronic mechanical irritation, mucosal diseases, nutritional deficiencies, systemic illness, polypharmacy, and declines in oral hygiene due to physical or cognitive limitations [86,87].

Histologically, aging is characterized by structural deterioration of the oral mucosa, including tissue dehydration and collagen destabilization [88], epithelial thinning, increased subepithelial collagen deposition, reduced elastin content, diminished epithelial ridges, and a flatter epithelial–connective tissue interface, all of which impair mucosal regenerative capacity [89,90]. Age-associated accumulation of senescent cells further disrupts epithelial turnover, contributing to reduced elasticity, impaired wound healing, and increased susceptibility to injury [91,92]. Elevated ROS levels compromise enamel by reducing its organic matrix, increasing susceptibility to fractures and cracks [93]. Dentin also undergoes age-related sclerosis as senescent odontoblasts deposit secondary dentin, diminishing sensory function and narrowing the pulp chamber [94]. These odontoblasts exhibit a shift from columnar to cuboidal morphology and accumulate lipofuscin granules with age [95]. Gingival recession is another frequent consequence of aging [89,96]. Collectively, these structural and functional changes, exacerbated by salivary gland hypofunction and oxidative stress, predispose older adults to oral mucosal diseases such as lichen planus, leukoplakia, recurrent aphthous ulcers, and burning mouth syndrome [97]. Impairment of epithelial barrier function, heightened pro-inflammatory cytokine production, and diminished antioxidant defenses further sustain chronic inflammation and hinder mucosal repair [98]. Senescent mesenchymal stem cells and T cells, along with their SASP profiles, contribute to the pathogenesis of oral lichen planus [99,100], while both senescence and dysregulated mitophagy play roles in the progression of oral leukoplakia [101].

Transcriptomic analyses of aged gingival and alveolar tissues demonstrate elevated DNA damage markers and enhanced SASP expression, reinforcing the mechanistic role of senescent cells in oral tissue degeneration [102,103].

SASP-associated matrix metalloproteinases (MMP-1, MMP-3, MMP-9) drive extracellular matrix remodeling by degrading collagen and elastin, weakening connective tissues and the periodontal ligament, which clinically manifests as gingival recession and reduced alveolar bone density [104].

Microbial dysbiosis is an important extrinsic stressor that promotes pro-senescent signaling in the oral cavity. Periodontal pathogen-derived lipopolysaccharide (LPS) and related microbial components have been shown to induce senescence-associated features in epithelial and immune cells, contributing to a feed-forward inflammatory environment in which dysbiosis sustains SASP-like signaling and chronic low-grade inflammation (inflammaging) [60,62,103].

Age-related immune senescence additionally compromises both innate and adaptive responses, increasing vulnerability to infections, promoting inflammaging, and contributing to degenerative alveolar bone loss [23,105,106,107,108,109,110].

Together, these findings highlight the significance of senescent cell accumulation in driving molecular, structural, and functional declines throughout the oral cavity and underscore the therapeutic potential of senolytics and SASP-modulating strategies.

### 3.2. Chronic Inflammation, Alveolar Bone Loss, and Periodontal Breakdown

Chronic inflammation is central to the pathogenesis of periodontal disease, and accumulating evidence implicates cellular senescence as a key mediator linking persistent microbial challenge to progressive alveolar bone destruction. Increased gene expression of senescence markers was detected in gingival tissues affected by periodontal disease in both humans and mice [111]. Senescent cells in periodontal tissues secrete SASP-associated cytokines, including IL-1β, IL-6, IL-8, and TNF-α, that recruit and dysregulate immune cells, perpetuating unresolved inflammation and destabilizing the balance between tissue degradation and repair [112,113,114,115]. These SASP factors can also induce paracrine senescence in neighboring periodontal cells, further reducing their regenerative capacity and accelerating clinical attachment loss.

**Figure 1:**
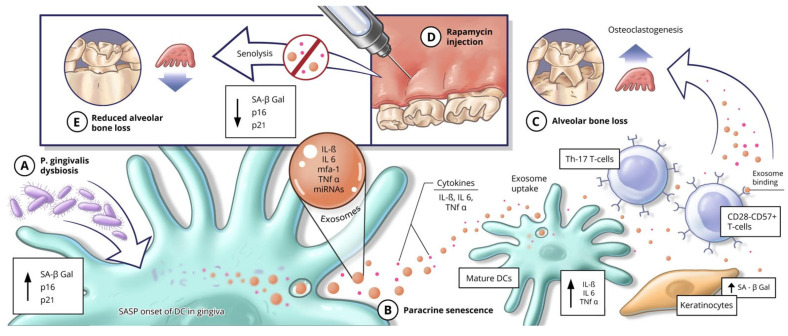
Proposed mechanism linking *Porphyromonas gingivalis*–induced dysbiosis, dendritic cell senescence, SASP exosomes and alveolar bone loss. (**A**) Oral dysbiosis driven by *P. gingivalis* promotes the onset of senescence in gingival dendritic cells (DCs), as indicated by increased senescence markers and acquisition of a senescence-associated secretory phenotype (SASP). (**B**) Senescent DCs release pro-inflammatory cytokines and exosomes enriched with inflammatory mediators and microRNAs, which propagate paracrine senescence in neighboring immune and epithelial cells, leading to DC maturation and amplification of local inflammation. Exosome uptake by recipient cells further enhances inflammatory signaling and cellular senescence. (**C**) These processes promote T-cell senescence and Th17 polarization, osteoclastogenesis, and progressive alveolar bone loss. (**D**) Local administration of rapamycin reduces senescence burden in gingival tissues, suppresses SASP signaling, and attenuates inflammatory cascades. (**E**) Clearance of senescent cells ultimately limits osteoclastogenesis and preserves alveolar bone integrity. Figure printed with permission from Augusta University and Peter Naktin.

In periodontal disease, keystone pathogens such as *Porphyromonas gingivalis* induce cellular senescence across multiple oral cell types, including fibroblasts [116], T cells and dendritic cells [24], alveolar osteocytes [62], while *Fusobacterium nucleatum* promotes senescence in fibroblasts [117] and keratinocytes [60]. In this context, pathogen-driven senescence is closely linked to the emergence of a SASP that sustains chronic inflammation and disrupts immune homeostasis (Figure 1). Metabolic stress further intensifies this process; hyperglycemia induces senescence in gingival macrophages [67], contributing to immune dysfunction and heightened inflammatory output. SASP factors released by senescent immune and stromal cells thereby act both as a consequence and a driver of periodontal pathology, perpetuating tissue destruction and impaired host defense [118].

Beyond the oral cavity, chronic *P. gingivalis* exposure has been shown in murine and in vitro models to induce senescence in dendritic cells with altered immune function and enhanced release of extracellular vesicles containing bacterial antigens and inflammatory mediators that amplify SASP signaling, impair immune surveillance, and accelerate alveolar bone resorption in experimental models [23,24]. These vesicles have been reported to traverse the blood–brain barrier and promote microglial activation and paracrine senescence, providing a potential mechanistic framework linking chronic periodontal infection to neuroinflammatory changes [119]. Consistent with this emerging model, independent epidemiological and experimental studies have detected *P. gingivalis* DNA and virulence factors, including gingipains and outer membrane vesicles, in the brains of individuals with Alzheimer’s disease and in animal models, where they correlate with microglial activation, amyloid pathology, and tau alterations [120,121]. Experimental periodontitis models further indicate that *P. gingivalis* components can disrupt blood–brain barrier integrity and induce neuroinflammatory signaling in vivo, with periodontitis models exhibiting enhanced immune cell infiltration and tau hyperphosphorylation [119,122]. Importantly, while these independent studies support the ability of periodontal pathogens and their vesicles to access the central nervous system and disrupt blood–brain barrier integrity, the specific contribution of oral pathogen-induced immune senescence to these processes remains an emerging concept that requires further validation across independent experimental and clinical models.

Collectively, these findings support a model in which pathogen-induced cellular senescence and SASP-mediated immune dysregulation act as central drivers of both local periodontal destruction and systemic inflammatory responses. Because cellular senescence sustains inflammation while impairing tissue regeneration, therapeutic strategies targeting senescent cells or their secretory outputs, including senolytics, senomorphics, and approaches aimed at modulating SASP-associated extracellular vesicles are increasingly being explored as promising avenues to disrupt this pathogenic cycle and preserve periodontal and systemic tissue integrity.

Senescence also influences outcomes in endodontics and orthodontics. Senescent dental pulp cells exhibit reduced proliferation and migration, and their SASP impairs tissue repair, compromising conservative root canal therapy [123,124,125]. In orthodontics, mechanical forces can modulate senescence in a context-dependent manner: human periodontal ligament [PDL] cells exposed to orthodontic treatment show reduced SA-β-Gal activity and increased α-klotho expression [126], whereas excessive mechanical stress induces senescence in PDL cells and cementoblasts, contributing to root resorption [70]. These findings highlight the challenges of managing senescence in patients requiring extensive orthodontic movement or in older individuals with higher baseline senescent cell accumulation.

Alveolar bone loss, a hallmark of both aging and periodontitis, reflects dysregulated bone remodeling driven by senescence within osteoblast, osteoclast, and osteocyte lineages in the alveolar bone. Senescent osteocytes and osteoblasts further impair bone homeostasis by suppressing osteogenesis and promoting osteoclast activity, thereby accelerating age-related bone loss. With aging, cumulative stressors reduce osteogenic potential and increase susceptibility to bone resorption [62,107,108,127,128].

A study in non-human primates demonstrated that periodontitis is marked by pro-osteoclastic gingival gene expression independent of age, whereas healthy gingiva exhibits an age-related shift toward a bone-destructive transcriptional profile [129]. Ebersole et al. further emphasized that biological aging, rather than chronological age, shapes the contribution of cellular senescence to periodontal disease progression [130].

Long-term exposure to bacterial products such as lipopolysaccharide (LPS), together with the accumulation of senescent osteocytes, promotes a pro-inflammatory SASP characterized by IL-6, IL-8, IL-1β, TNF-α, and various proteases, intensifying periodontal disease severity [108].

SASP factors and matrix-degrading enzymes from senescent cells disrupt the local microenvironment, promote dysbiosis, and propagate senescence to neighboring cells, contributing to chronic periodontal inflammation. Consequently, osteoblastic and osteoclastic activities become increasingly imbalanced with age, while the immunomodulatory, migratory, and differentiation capacities of mesenchymal progenitor cells decline, collectively driving enhanced alveolar bone loss [115,131].

Senescence similarly impairs the regenerative capacity of dental pulp stem cells (DPSCs), limiting their osteogenic and odontogenic potential [132,133,134]. Notably, removal of senescent cells has been shown to restore regenerative function in both dental pulp and periodontal ligament stem cell populations [132,135].

### 3.3. Senescent Cell Accumulation in Oral Carcinogenesis

Cellular senescence plays a paradoxical role in oral carcinogenesis, particularly in OSCC. In its acute form, senescence serves as a tumor-suppressive barrier that halts the proliferation of cells experiencing oncogenic or genotoxic stress [136,137]. However, when senescent cells persist, their SASP profiles generate a pro-tumorigenic microenvironment that facilitates the progression from dysplasia to invasive carcinoma. SASP factors, including IL-6, IL-8, VEGF, and matrix-degrading enzymes, promote epithelial–mesenchymal transition (EMT), angiogenesis, invasion, and extracellular matrix remodeling [49,138].

Chronic SASP-driven inflammation recruits immune cells that paradoxically support tumor expansion by fostering immunosuppression, cytokine-driven proliferation, and stromal remodeling [139]. Senescent stromal fibroblasts in the OSCC microenvironment further enhance cancer cell invasion and metastatic potential through secretion of growth factors and chemokines [45,140,141]. Additionally, senescent cells can evade immune clearance, perpetuating their tumor-supportive influence [142].

The dual nature of senescence in OSCC underscores the need for nuanced therapeutic approaches. Strategies such as selective clearance of senescent cells after chemotherapy (to eliminate therapy-induced senescence), SASP inhibition, and modulation of stromal senescence are being investigated for their potential to improve treatment outcomes [45,143]. Targeting the senescent cell niche may therefore represent a novel avenue to disrupt OSCC progression and enhance antitumor immunity.

### 3.4. Salivary Gland Dysfunction and Oral Dryness

With age, salivary glands undergo metabolic disruption and structural remodeling, including acinar cell loss and replacement with fibrous and adipose tissue, which directly impair secretory function [144,145]. Clinically, these alterations correspond with significantly reduced unstimulated and stimulated salivary flow in older adults, who also exhibit a high prevalence of dry mouth symptoms [146]. Aging also induces compositional changes in saliva, including altered electrolyte levels, reduced antioxidant enzyme activity (peroxidase, glutathione peroxidase, catalase), and decreased mucin content (MUC1, MUC2, MUC7) [144]. These functional and compositional changes increase susceptibility to oral inflammation, burning mouth syndrome, and certain oral cancers, reflecting the impact of salivary gland senescence and xerostomia [144,147,148,149].

Senescence-associated persistent inflammatory burden exerts a deleterious effect on salivary tissues, where synergistic cytokine signaling (e.g., IL-6, TNF-α) and oxidative stress drive glandular fibrosis and inhibit resident stem cells [110,145]. The consequent impairment of regenerative capacity results in acinar atrophy and fatty degeneration, leading to reduced secretory function [145]. Clinically, this dysfunction manifests not only as xerostomia but also as a distinct susceptibility to root-surface caries, opportunistic fungal infections (such as candidiasis), and mucosal fragility due to the loss of protective salivary glycoproteins [150].

Senescence also affects oral responses to cancer therapy. CIS in head and neck squamous cell carcinoma can contribute to therapy resistance [52], while radiation therapy induces senescence in salivary gland cells, resulting in hyposalivation [54,55] and subsequent oral complications, including difficulty eating and dental caries [151].

Radiation therapy for head and neck cancer induces irreversible damage through senescence of ductal and progenitor cells. In murine models, the senolytic agent Navitoclax (ABT-263) selectively eliminated p16^Ink4a+^ senescent cells after irradiation, restoring salivary flow, acinar regeneration, and organoid formation from surviving stem/progenitor cells [54]. However, systemic BCL-XL inhibition by Navitoclax caused transient thrombocytopenia [152,153], highlighting the need for localized delivery strategies to minimize hematologic toxicity.

Aging in murine models is also associated with reduced expression of key functional markers such as aquaporin-5 and amylase, along with decreased progenitor cell markers including c-Kit and cytokeratin-5, indicating diminished regenerative capacity [154]. Senescent cells accumulate in salivary glands with age and chronic injury, secreting a SASP rich in pro-inflammatory cytokines, chemokines, and matrix-remodeling enzymes. This secretome contributes to fibrosis, extracellular matrix deposition, and loss of acinar integrity [54,145]. SASP factors alter tight-junction proteins such as claudin-3 and occludin, impairing ductal function and reducing saliva secretion. D-galactose-induced aging models further demonstrate that senescent-cell accumulation correlates with acinar atrophy, tight-junction disorganization, and decreased salivary output, effects that can be partially reversed by stem cell-derived exosomes [155]. In primary Sjögren’s syndrome, basal ductal progenitor cells exhibit elevated p16^INK4A^ and other senescence markers, which associate with reduced salivary flow, glandular architectural disruption, and lymphocytic infiltration [156,157]. Oxidative stress, chronic inflammation, and immune dysregulation further accelerate senescence of acinar and ductal cells, compounding secretory impairment [145,158].

Collectively, these findings establish senescence as a potential mechanistic driver of salivary gland hypofunction and xerostomia. By promoting chronic inflammation, fibrosis, epithelial barrier dysfunction, and progenitor-cell exhaustion, senescence produces a self-reinforcing decline in glandular secretory capacity. Emerging senotherapies, including senolytics, SASP-modulating agents, and stem cell-derived exosomes, have shown promise in restoring salivary gland structure and function in aging models, highlighting the potential of senescence-targeted interventions as transformative therapies for age-related oral dryness and salivary gland disease.

**Table 2:** Senescence in Oral Diseases: Senescent Cell Types, Hallmark Markers, SASP Profiles, and Functional Consequences.

Oral Condition/Disease	Senescent Cell Types	Hallmark Senescence Markers	Key SASP Components	Functional Consequences	References
Chronic Periodontitis	Gingival fibroblasts, epithelial cells, macrophages, dendritic cells, osteocytes	p16, p21, γ-H2AX, SA-β-Gal, ↓ Lamin B1	IL-6, IL-1β, TNF-α, MMP-1/3, GM-CSF, RANKL	Persistent inflammation, ECM breakdown, alveolar bone loss, paracrine spread of senescence	[23,24,62,107,108,109,110,111,112,113,114,115]
Diabetes-associated periodontitis	Macrophages, endothelial cells, fibroblasts	p16, p21, ROS-linked DNA damage	IL-1β, IL-6, IL-18, TNF-α, CXCLs	Exacerbated periodontal inflammation, impaired healing, microvascular dysfunction	[64,65,66,67]
Alveolar bone loss/skeletal aging	Osteocytes, osteoblasts	p16, p21, SA-β-Gal, telomere shortening, γ-H2AX	IL-6, IL-8, RANKL, MMP-13	Reduced bone formation, osteoclast activation, compromised structural integrity	[62,107,108,127,128].
Oral mucosal aging	Keratinocytes, fibroblasts	p16, p21, SA-β-Gal	IL-6, GM-CSF, MMPs, CXCL8	Thinning epithelium, delayed wound healing, increased susceptibility to injury	[88,90,91,92,102,103]
Dental pulp aging/pulpitis	Pulp fibroblasts, odontoblasts	p16, p21, SA-β-Gal, SAHF	IL-6, IL-1β, MCP-1, MMPs	Decreased regenerative potential, chronic inflammation, impaired dentin repair	[132,133,134]
Orthodontic root resorption	PDL fibroblasts, cementoblasts, osteocytes	p16, p21, γ-H2AX	RANKL, IL-6, IL-11	Inflammation-driven resorption, altered bone/PDL remodeling	[68,70]
Salivary gland dysfunction (aging or radiation-induced)	Ductal epithelial cells, progenitor cells, acinar cells	p16, p21, γ-H2AX	IL-6, IL-8, CCL chemokines, MMPs	Xerostomia, glandular fibrosis, loss of regenerative capacity, replacement of acinar cells with fibrous and adipose tissue	[54,55,144,145]
Oral potentially malignant disorders (OLP, Leukoplakia, dysplasia)	Dysplastic keratinocytes, fibroblasts	p16, p21, SA-β-Gal, OIS markers	IL-6, IL-8, MMPs, VEGF	Early tumor-suppressive barrier but SASP promotes local proliferation and immune infiltration	[43,82,100,101]
Oral squamous cell carcinoma (OSCC)	CAFs, senescent tumor cells (therapy-induced)	p16, p21, SA-β-Gal, DDR markers	IL-6, IL-8, TGF-β, VEGF, MMP-2/9	Invasion, angiogenesis, immune suppression, therapy resistance	[45,49,138,139,140,141,142]
Microbial-driven immune senescence (Pg, Fn, OMVs)	Dendritic cells, macrophages, epithelial cells	p21, γ-H2AX, mitochondrial stress markers	IL-1β, IL-6, TNF-α, IFN-associated SASP	Systemic immunosenescence, altered T-cell responses, enhanced periodontal inflammation	[24,60,62,116,117]

Senescence in Oral Diseases: Senescent Cell Types, Hallmark Markers, SASP Profiles, and Functional Consequences. ↓, Decrease; ECM, Extracellular matrix; SA-β-Gal, Senescence-associated β-Galactose; ROS, reactive oxygen species; RANKL, Receptor Activator of Nuclear factor Kappa-B Ligand; MMP, Matrix metalloproteinase; GM-CSF, Granulocyte-Macrophage Colony-Stimulating Factor; PDL, Periodontal ligaments; OLP, Oral Lichen Planus; VEGF, Vascular endothelial growth factor; SASP, Senescence-Associated Secretory Phenotype; DDR, DNA damage response; Pg, Porphyromonas gingivalis; Fn, Fusobacterium nucleatum; OMVs, Outer membrane vesicles.

## 4. Targeting Senescence in Oral Disease Management

Cellular senescence, particularly when senescent cells persist in oral tissues, has emerged as a potential contributor to the pathogenesis of chronic oral diseases. SASP drives a self-sustaining cycle of inflammation, tissue degradation, and dysregulated bone metabolism. By targeting these maladaptive processes, novel therapeutic strategies aim not only to alleviate symptoms but also to modify disease progression.

While early strategies focused on repurposing systemic oncology drugs, the field is rapidly shifting toward precision medicine. Current approaches prioritize local delivery, tissue-specific targeting, and next-generation therapies, including senolytics, senomorphics, immune modulation, and gene- or cell-based interventions, to restore tissue homeostasis and prevent oral pathologies such as periodontitis, alveolar bone loss, and mucosal disorders, while minimizing the toxicity of broad-spectrum agents [108].

### 4.1. Senolytic Therapies: Selective Elimination of Senescent Cells

Senolytic therapies aim to selectively induce apoptosis in senescent cells (“senolysis”), thereby removing the source of chronic inflammation and fostering regeneration. Targeted depletion of senescent cells in prematurely aged mice improved exercise performance and increased muscle fiber diameter [159]. Senolytic therapies represent a novel approach in oral disease management, aimed at selectively eliminating senescent cells that accumulate in periodontal and mucosal tissues. Preclinical studies using the p16-3MR transgenic mouse model demonstrate that conditional ablation of p16^INK4a^-positive senescent cells alleviates local inflammation and significantly reduces alveolar bone loss in experimental periodontitis, directly implicating senescent cells in disease pathogenesis [109].

Pharmacological senolytics, particularly the combination of Dasatinib and Quercetin (D + Q), have shown considerable potential. Dasatinib is a tyrosine kinase inhibitor and Food and Drug Administration–approved agent for leukemia [160,161], while Quercetin is a plant-derived flavonoid with antioxidant, anti-inflammatory, and antiaging properties, modulating pathways including NF-κB, PI3K, and Bcl cascades [162,163,164]. In murine periodontitis models, D + Q treatment reduced gingival senescence, attenuated SASP burden, and preserved periodontal architecture [165].

Age-related degeneration of the temporomandibular joint (TMJ) has also been linked to the accumulation of senescent chondrocytes and fibrocartilage cells, which secrete SASP factors such as IL-6 and MMP-13, contributing to cartilage thinning and subchondral bone erosion [166,167]. Treatment of aged mice with D+Q reduced senescent cell burden in TMJ tissues, decreased SASP factor expression, and improved cartilage thickness, proteoglycan content, and subchondral bone structure, highlighting the potential of senolytics to mitigate TMJ degeneration [168]. However, direct evidence of full SASP secretion by senescent TMJ chondrocytes or fibrocartilage cells with lineage tracing remains limited.

Senescence also impairs the regenerative potential of dental pulp stem cells (DPSCs). Exposure to oxidative stress (e.g., H_2_O_2_) or prolonged culture induces senescence in DPSCs, marked by SA-β-gal positivity and upregulation of p16/p21, leading to loss of osteo/odontogenic potential [133,134]. Treatment with the BCL-2/BCL-XL inhibitor ABT-263 restores proliferation and differentiation, indicating that senescence drives functional decline in DPSCs [132]. Senescent keratinocytes are implicated in periodontal diseases. In preclinical studies, D+Q treatment of human gingival keratinocytes exposed to *F. nucleatum* reduced senescence markers (p16, SA-β-gal, Lamin B1 decline) and SASP-associated inflammatory mediators (IL-8, MMP-1, MMP-3) [165]. Oral D + Q administration in aged mice similarly reduced gingival senescence, mitigated naturally progressing alveolar bone loss, decreased neutrophil infiltration, and improved periodontal ligament and bone metabolism parameters [165,169]. Some benefits of Quercetin may derive from its ability to lower senescent cell burden, reduce oxidative stress, dampen inflammation, and promote a healthier oral microbial environment [170]. While these preclinical findings are promising, clinical evidence in humans is lacking. No trials have yet demonstrated that D+Q or other senolytics improve periodontal disease outcomes.

The off targets of senolytics limit immediate clinical translation. D+Q may cause gastrointestinal disturbances and immunomodulatory changes, while Navitoclax carries a thrombocytopenia risk due to BCL-XL inhibition in platelets [171]. Systemic senolysis can also perturb host–microbiome interactions; for example, D+Q treatment in aged mice altered gut microbial diversity and reduced immune responsiveness to influenza infection [172,173]. This limits their utility for non-life-threatening oral conditions.

The most promising “next-generation” solution lies in localized and smart delivery systems [174]. This includes local depots and hydrogels, senolytic nanoparticles and proteolysis-targeting chimeras. For example, encapsulated senolytics (e.g., ABT-263 or D+Q) in biodegradable PLGA microspheres or thermosensitive hydrogels have been investigated as antisenescence dosage forms for intravertebral Disc Degeneration and showed promising results [175,176]. They have not yet been investigated in oral pathology, but local dosage forms of Navitoclax or D+Q will enable sustained release within the periodontal pocket or TMJ space. This strategy maximizes local efficacy while minimizing systemic exposure. Among emerging approaches, smart senolytic nanoparticles and proteolysis-targeting chimera (PROTAC)-based senolysis appear particularly promising due to their specificity and safety profiles. For instance, mesoporous silica nanoparticles coated with galacto-oligosaccharides, as “smart” nanoparticles, can preferentially target cells with high SA-β-gal activity, releasing their cytotoxic payload only after internalization by a senescent cell [177]. PROTACs can degrade specific senescence-essential proteins (like Bcl-xL) rather than just inhibiting them. This technology offers higher specificity and lower toxicity than traditional small molecule inhibitors [178]. The precise oral cell populations that benefit most from senolytic clearance, as well as potential unintended effects on protective or reparative responses, remain to be fully elucidated.

### 4.2. Senomorphic Agents: Modulating the SASP and Pathways of Senescence

Senomorphic agents selectively suppress the detrimental senescence-associated secretory phenotype (SASP) without eliminating senescent cells, thereby preserving beneficial functions such as tissue repair while mitigating chronic inflammation. Rapamycin, a well-studied mTOR inhibitor, exemplifies this approach. In aged murine periodontium, rapamycin reduced alveolar bone loss [179] and downregulated SASP-associated cytokines, including IL-6, IL-1β, and TNF-α, via modulation of mTOR and NF-κB signaling pathways [24,180,181].

In vitro studies further support these effects. In human periodontal ligament stem cells (PDLSCs) exposed to inflammatory stress, rapamycin suppressed senescence and restored osteogenic differentiation through the PI3K/AKT/mTOR axis [182]. Similarly, rapamycin attenuated immune senescence in dendritic cells infected with *P. gingivalis* and reduced the release of SASP-associated exosomes [23]. Rapamycin’s benefits span multiple periodontal tissues and mechanisms [181]. It enhances protective autophagy by increasing LC3-II, Beclin-1, and ULK1, improving cellular resilience [183,184]; reduces inflammatory cytokines while boosting regulatory T-cell activity, creating a less destructive immune environment; promotes osteogenesis by elevating ALP, Runx2, BMP-2, and OCN while suppressing RANKL [185]; mitigates cellular senescence in gingival tissues by decreasing p16^INK4a^ and SA-β-gal [23,24]; and shifts the oral microbiome toward a youthful, health-associated profile [179]. Despite these benefits, rapamycin may have context- or dose-dependent limitations. Excessive autophagy can impair tissue repair [186]; its effects on bone-regulatory molecules such as OPG and COL1 may be inconsistent [187,188,189]; and epithelial wound closure can be delayed under some conditions [190]. Overall, rapamycin demonstrates strong therapeutic potential for periodontal health, though optimal use requires careful calibration of dose, timing, and tissue-specific responses. Collectively, senomorphics offer dual advantages: limiting chronic inflammation while preserving regenerative capacity in oral tissues.

### 4.3. Enhancing Immune-Mediated Senescent Cell Clearance

Another frontier involves leveraging the body’s innate and adaptive immune systems to clear senescent cells. Under physiological conditions, senescent cells are surveilled and eliminated by natural killer (NK) cells, macrophages, and cytotoxic T cells. However, with aging and chronic inflammation, immune clearance wanes, permitting senescent cells to accumulate and perpetuate dysfunction [191]. Reinvigorating immune responses has shown promise in preclinical models. For example, enhancing NK cell cytotoxicity improved clearance of senescent fibroblasts and reduced SASP-mediated inflammation [192].

Likewise, senescent cells evade macrophage phagocytosis through upregulation of the “don’t-eat-me” signal CD47 and the modifying enzyme QPCT/L. Blocking this axis restores macrophage-mediated clearance and reduces inflammatory burden, suggesting a therapeutic opportunity for oral disease contexts such as periodontitis. These strategies, when combined with pharmacological senolytics, may achieve synergistic elimination and immune surveillance of senescent cells, offering durable disease control [193].

### 4.4. Prophylactic Strategies to Delay Senescence Onset in Oral Tissues

Beyond clearance, delaying the onset of senescence itself represents an essential prophylactic approach. Chronic oxidative stress, microbial dysbiosis, and repetitive mechanical strain all accelerate premature senescence in periodontal fibroblasts, gingival epithelia, and stem cells. Interventions that target these upstream drivers can extend the functional lifespan of oral cells. For instance, metformin has been shown to prevent oxidative stress-induced senescence in human periodontal ligament cells, sustaining both proliferation and regenerative capacity [194].

In vivo, short-term rapamycin therapy rejuvenated the aged oral cavity of mice, reversing periodontal bone loss, reducing inflammation, and restoring a youthful oral microbiome within just eight weeks. Together, these findings emphasize that lifestyle- and drug-based interventions can act as early modifiers of cellular aging, complementing senolytic and senomorphic therapies to preserve oral health across the lifespan [179,195].

### 4.5. Gene and Cell-Based Interventions to Modulate Senescence

Cutting-edge gene and cell-based strategies are expanding the therapeutic arsenal against oral tissue senescence. Genetic targeting of canonical regulators such as p16^INK4a^ and p53/p21 has revealed that selective ablation of senescent cells can curb inflammation and protect alveolar bone in experimental models of periodontitis [109].

Cell-based approaches focus on rejuvenating mesenchymal stem cells (MSCs), including PDLSCs, which often lose regenerative potential under senescent stress. Rapamycin treatment has been shown to rescue osteogenic differentiation of PDLSCs under oxidative conditions, while genetic modification strategies such as hTERT overexpression have extended MSC proliferative lifespan and maintained osteogenic potential. These approaches highlight the feasibility of ex vivo rejuvenation for transplantation therapies [194,196]. Moreover, immunogenetic strategies such as senolytic CAR T cells have demonstrated the ability to selectively target and eliminate senescent cells in preclinical models, reversing fibrosis and metabolic dysfunction [197]. Engineering Chimeric Antigen Receptor (CAR) T cells to recognize senescent-cell-specific surface proteins (e.g., uPAR) is being explored as a targeted approach to selectively eliminate senescent cells. In models of liver fibrosis, these cells successfully ablated senescent tissues and reversed pathology [198]. Although not yet applied directly to oral disease, these technologies illustrate the potential of precision cellular therapies to restore oral tissue homeostasis in the future.

Targeting senescence in oral disease management has moved from conceptual framework to translational promise, with a growing body of preclinical evidence highlighting the potential of senolytics, senomorphics, immune-modulating strategies, and gene- or cell-based interventions. While most findings to date are derived from animal models and in vitro studies, they collectively provide a strong rationale for moving toward early-phase clinical trials in humans.

A central challenge lies in achieving a therapeutic balance: eliminating or suppressing detrimental aspects of senescence while preserving its beneficial roles in wound healing, tissue remodeling, and tumor suppression. Combining approaches such as senolytic clearance with immune rejuvenation or senomorphics with prophylactic interventions may offer the most effective long-term strategy. Additionally, advances in precision medicine, including targeted drug delivery, biomarker discovery, and patient-specific cell therapies, hold promise for tailoring interventions to individual disease contexts.

## 5. Conclusions and Future Research

Cellular senescence, driven by the accumulation of senescent cells and their SASP, plays a significant role in oral aging and disease, contributing to periodontitis, alveolar bone loss, mucosal deterioration, and radiotherapy-induced salivary gland dysfunction. Targeting senescent cells with senolytic agents represents a promising therapeutic strategy, yet oral applications remain in their early stages and face significant challenges. These observations highlight the need for tissue-targeted delivery strategies to confine senolytic activity to the oral cavity.

Looking forward, advancing oral senotherapy will require the development of a comprehensive oral senescence atlas to map cell-type-specific markers, such as p16^INK4a^, p21, SASP components, and chromatin signatures, across gingiva, periodontium, dental pulp, and salivary glands. Integration of single-cell and spatial transcriptomics data could inform targeted, localized delivery systems and rational combination therapies coupling senolytics with senomorphics. Future studies should explore low-dose, controlled-release, or site-specific formulations that minimize systemic exposure while assessing long-term outcomes, including alveolar bone preservation, mucosal integrity, and microbiome homeostasis. Through such precision approaches, senolytic and senomorphic therapies have the potential to transition from experimental promise to clinically viable interventions in oral medicine.

## Data Availability

No new data were created or analyzed in this study. Data sharing is not applicable to this article.
